# MicroRNA-mRNA expression profiles and their potential role in cadmium stress response in *Brassica napus*

**DOI:** 10.1186/s12870-019-2189-9

**Published:** 2019-12-19

**Authors:** Ying Fu, Annaliese S. Mason, Yaofeng Zhang, Baogang Lin, Meili Xiao, Donghui Fu, Huasheng Yu

**Affiliations:** 1 Institute of Crop and Nuclear Technology Utilization, Zhejiang Academy of Agricultural Sciences, Hangzhou, China; 2 Department of Plant Breeding, IFZ for Biosystems, Land Use and Nutrition, Justus Liebig University, Heinrich-Buff-Ring 26-32, 35392 Giessen, Germany; 3 Key Laboratory of Crop Physiology, Ecology and Genetic Breeding, Ministry of Education, Agronomy College, Jiangxi Agricultural University, Nanchang, 330045 China

**Keywords:** *Brassica napus*, Cadmium, microRNAs, mRNAs, Expression

## Abstract

**Background:**

Oilseed rape is an excellent candidate for phytoremediation of cadmium (Cd) contaminated soils given its advantages of high biomass, fast growth, moderate metal accumulation, ease of harvesting, and metal tolerance, but the cadmium response pathways in this species (*Brassica napus*) have yet to be fully elucidated. A combined analysis of miRNA and mRNA expression to infer Cd-induced regulation has not been reported in *B. napus*.

**Results:**

We characterized concurrent changes in miRNA and mRNA profiles in the roots and shoots of *B. napus* seedlings after 10 days of 10 mg/L Cd^2+^ treatment. Cd treatment significantly affected the expression of 22 miRNAs belonging to 11 families in the root and 29 miRNAs belonging to 14 miRNA families in the shoot. Five miRNA families (MIR395, MIR397, MIR398, MIR408 and MIR858) and three novel miRNAs were differentially expressed in both tissues. A total of 399 differentially expressed genes (DEGs) in the root and 389 DEGs in the shoot were identified, with very little overlap between tissue types. Eight anti-regulation miRNA-mRNA interaction pairs in the root and eight in the shoot were identified in response to Cd and were involved in key plant stress response pathways: for example, four genes targeted by miR398 were involved in a pathway for detoxification of superoxide radicals. Cd stress significantly impacted the photosynthetic pathway. Transcription factor activation, antioxidant response pathways and secondary metabolic processes such as glutathione (GSH) and phenylpropanoid metabolism were identified as major components for Cd-induced response in both roots and shoots.

**Conclusions:**

Combined miRNA and mRNA profiling revealed miRNAs, genes and pathways involved in Cd response which are potentially critical for adaptation to Cd stress in *B. napus*. Close crosstalk between several Cd-induced miRNAs and mRNAs was identified, shedding light on possible mechanisms for response to Cd stress in underground and aboveground tissues in *B. napus*. The pathways, genes, and miRNAs identified here will be valuable targets for future improvement of cadmium tolerance in *B. napus*.

## Background

Cadmium (Cd) is one of the most serious major soil contaminants [1]. Cd is not an essential element for plants, but it can be readily absorbed by roots and transported to the above-ground organs [2]. Forty mg/L Cd in soil was already found to affect yield in some crops, while 200 mg/L had serious effects [3]. High Cd concentrations damage plant growth and reproduction through decreasing nutrient uptake, inhibiting photosynthetic activity [4], distorting membranes [5], and stunting plant growth [6], resulting in overall decreases in crop yield [2]. Cd accumulation in the human body over time poses serious health risks, including a risk of chronic toxicity in kidney tubules, bones, lungs and other organs [7].

Plants have evolved a range of mechanisms for heavy metal detoxification, including pumping heavy metals out of the plasma membrane, chelation, and binding to various thiol compounds in the cytosol followed by sequestration into vacuoles [8–10]. Antioxidant and signaling mechanisms also participate in the heavy metal detoxification process [11]. Glutathione (GSH) is a well documented and also highly essential component for Cd detoxification [12], and GSH- or phytochelatin (PC)-conjugated vacuolar sequestration is one of the most important mechanisms of Cd accumulation and tolerance in plants [13–15]. Many other key genes have also been also shown to be involved in Cd detoxification and tolerance in plants, such as *ZNT* [16], *HMA* [14], *NRAMP* [14], *ZntA* [17], *CAD* [18], *PDR* [19, 20], *PDR* [20], *ATM* [21], *ACBP* [22], *EIN* [23], *ABCC* [14], *ZIF* [14], *MTP* [14], *PCR* [14], and *MAN* [14]. Additionally, miRNAs play important roles in abiotic stress responses [24]. Cd-responsive miRNAs have been identified in rice (*Oryza* spp.) [25, 26], narrowleaf cattail (*Typha angustifolia*) [27], *Medicago truncatula* [28], and radish (*Raphanus* spp.) [29], strongly supporting miRNAs as key post-transcriptional regulators of Cd stress responses in plants [30].

Phytoremediation, where plants are used to remove heavy metals from soils, is an environmentally friendly, cost-effective technique for remediation of heavy metal-contaminated soils [31]. Oilseed rape (*Brassica napus*) is not only an important oil crop worldwide, but also an excellent candidate for phytoremediation given its advantages of high biomass, fast growth, moderate metal accumulation, ease of harvesting, and metal tolerance [32]. A number of studies have elucidated the physiological responses of *B. napus* to Cd stress, with identification of key Cd-stress-responsive genes *BnPCS1*, *BnGSTU12*, *BnGSTU5*, and *BnHMAs*. Three major studies to date have attempted to identify Cd-responsive miRNAs through genome-wide studies in *B. napus* [33–35]. Huang et al. (2010) found 58 miRNAs after small RNA sequencing of root, stem and leaf tissue after treatment with 80 μM Cd (about 15 mg/L) for 12, 24, 48 and 72 h, of which 9 miRNAs were differentially expressed as a result of Cd stress in at least one tissue type relative to the control [33]. Zhou et al. (2012) found 84 miRNAs in root and shoot tissue of young seedlings after 40 and 80 μM Cd treatment for 6–48 h, of which 13 miRNA families and five novel miRNAs were differentially expressed in the shoot and 8 miRNA families were differentially expressed in the root; at least 43 high-confidence gene targets of differentially expressed miRNAs in the roots and 126 convincing gene targets in the shoots were identified through degradome analysis [35]. Jian et al. (2018) looked at miRNA and mRNA expression in response to 1000 μM CdCl_2_ treatment (approximately 180 mg/L) in whole *B. napus* seedlings 0, 1 and 3 days after treatment, and identified 147 miRNAs, of which 39 were differentially expressed in response to Cd treatment, but for which target genes were only computationally predicted from the reference genome [34].

To date, studies have rarely profiled genome-wide miRNA and mRNA (gene expression) simultaneously under Cd stress. The optimal Cd concentration and treatment time with which to assess stress response is also still unclear. To acquire a deeper understanding of how miRNA might interact with mRNA to regulate response to Cd stress in *B. napus*, we sequenced miRNA and mRNA in root and shoot tissues of oilseed rape seedlings after an optimized Cd-stress treatment of 10 days at 10 mg/L and in controls, in order to help elucidate the molecular genetic mechanisms underlying oilseed rape response to Cd stress.

## Results

### Leaf chlorophyll content decreases in response to cd stress in *B. napus*

**Fig. 1:**
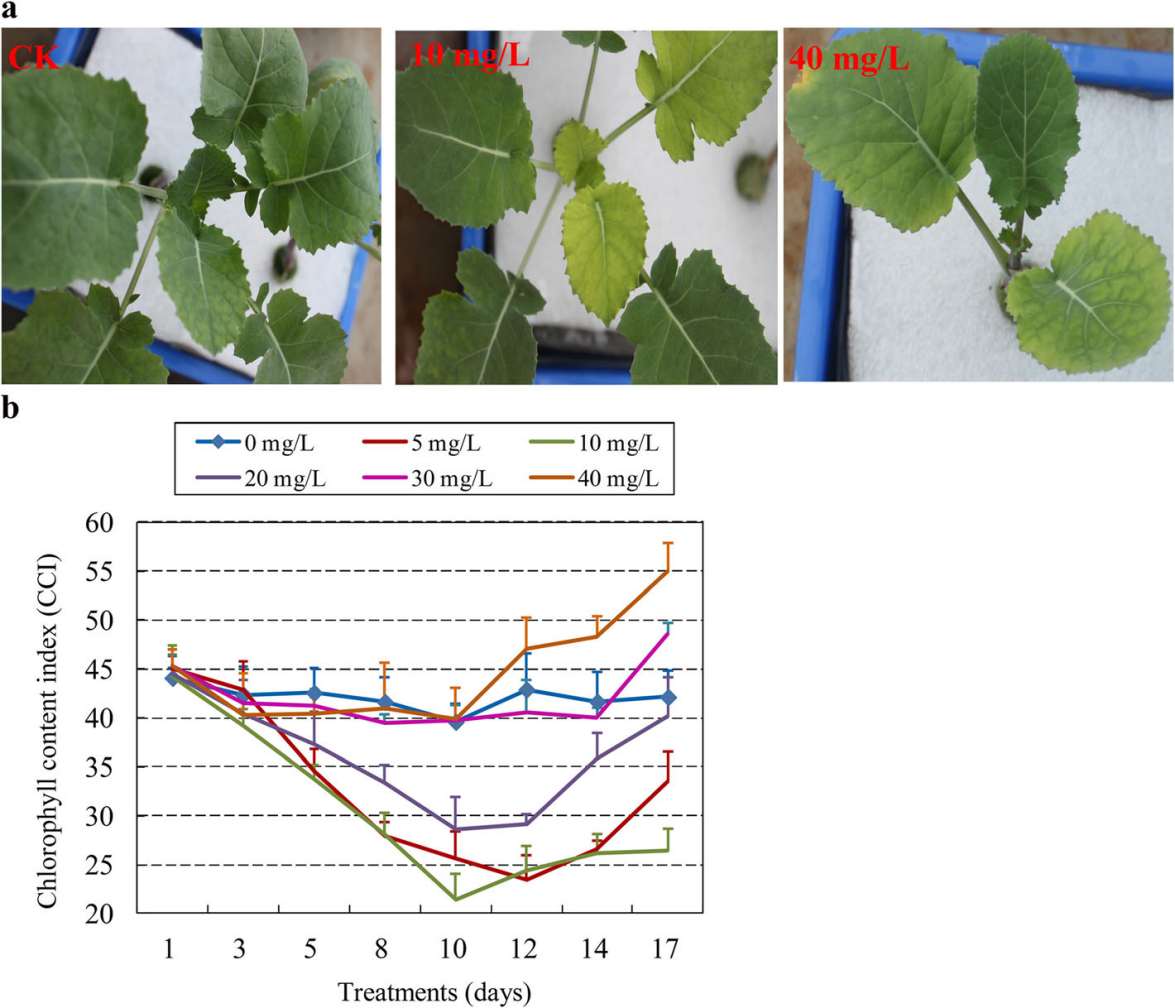
The growth performance and total chlorophyll content changes of *B. napus* seedlings exposed to six Cd concentration gradients. **a** The leaf chlorosis performance of *B. napus* seedlings exposed to no stress and to Cd stress under different Cd concentrations. **b** The changes in total chlorophyll content of *B. napus* seedlings exposed to six Cd^2+^ concentrations over time: 0 mg/L (control), 5 mg/L, 10 mg/L, 20 mg/L, 30 mg/L, 40 mg/L

Leaf chlorophyll content clearly indicated response to Cd stress (Fig. 1a). Across all Cd treatment concentrations leaf chlorophyll content generally decreased until about day 10, then started to increase again (Fig. 1b). Of the different Cd treatments, 10 mg/L Cd^2+^ for 10 days resulted in the maximum decrease in chlorophyll content. Thus, the seedlings treated with 10 mg/L Cd^2+^ for 10 days were used for analysis of miRNA and mRNA expression profiles in response to Cd stress.

### Expression changes of miRNAs are involved in the response to cd in oilseed rape

**Fig. 2:**
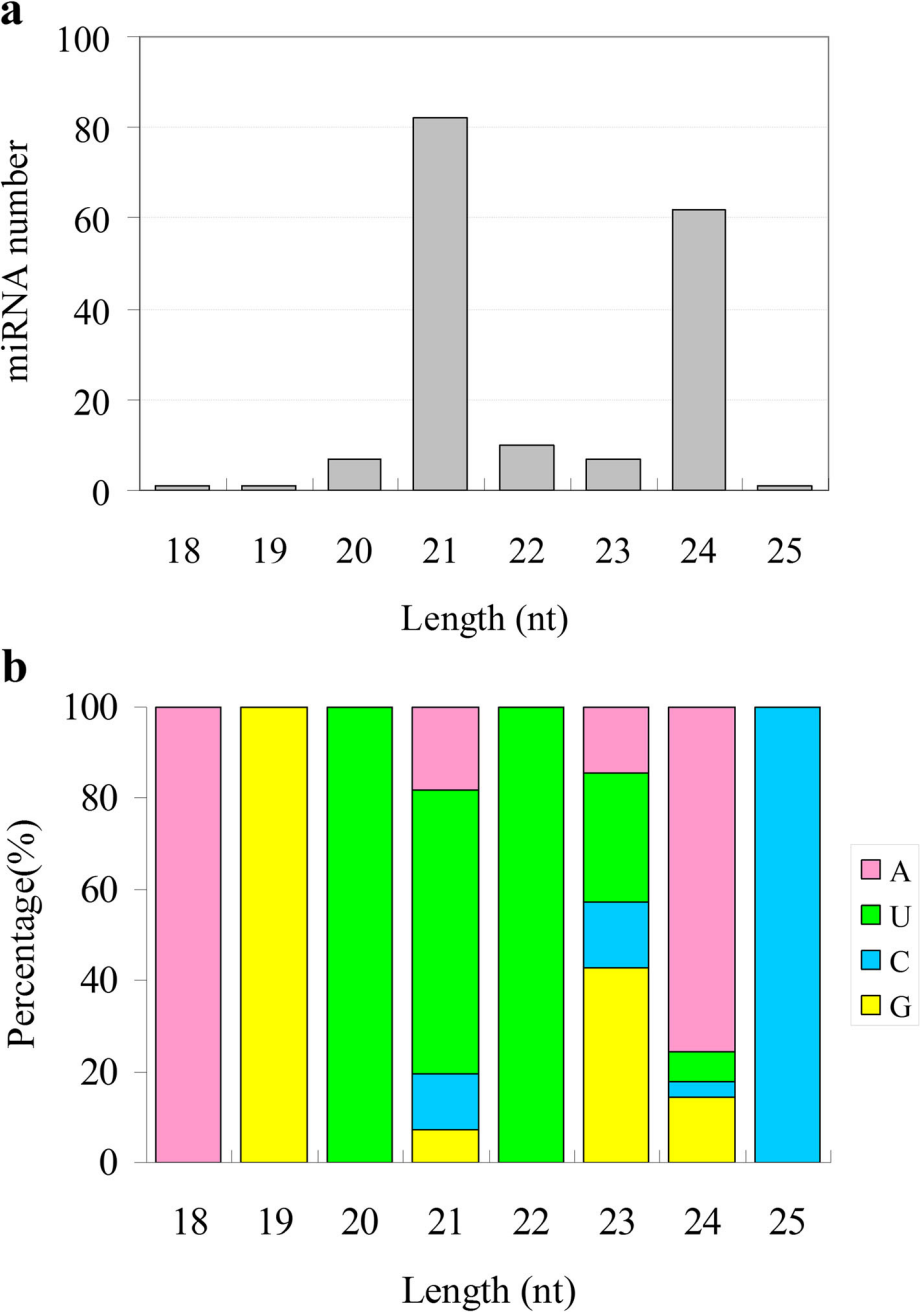
Lengths of small RNAs (sRNAs) and first base .s of mature miRNAs in 12 libraries (three biological replicates) constructed from shoot and root tissues of *Brassica napus* seedlings under 0 or 10 mg / mL cadmium stress for 10 days. **a** The proportion of sRNAs of different lengths in the 12 sequencing libraries. The Y-axis displays the number of sRNAs of a certain length, while the X-axis represents sRNAs of different lengths. **b** The first base preference of mature miRNAs in the 12 libraries. The Y-axis displays the proportion of mature miRNAs with a certain base type as the first base, and the X-axis represents the length classification of the sRNAs

We constructed 12 small RNA libraries (root control, root Cd treatment, shoot control, shoot Cd treatment; three biological replicates for each sample), which yielded 284 M raw reads, resulting in 239 M clean reads (Additional file 1: Table S2). A total of 171 miRNAs were identified. The length distribution of miRNAs ranged from 18 nt to 25 nt in the 12 small RNA libraries, of which the most abundant were 21 nt miRNAs, followed by 24 nt and 22 nt miRNAs (Fig. 2a). Mature 20–22 nt miRNAs mostly started with ‘U’ as the first base (62.2–100%), while 24 nt miRNAs mostly started with ‘A’ (75.8%) (Fig. 2b). Of the 171 miRNAs identified, 99 (57.9%) were previously known and belonged to 44 families. The 72 newly-predicted miRNAs are displayed in Additional file 1: Table S3.

**Fig. 3:**
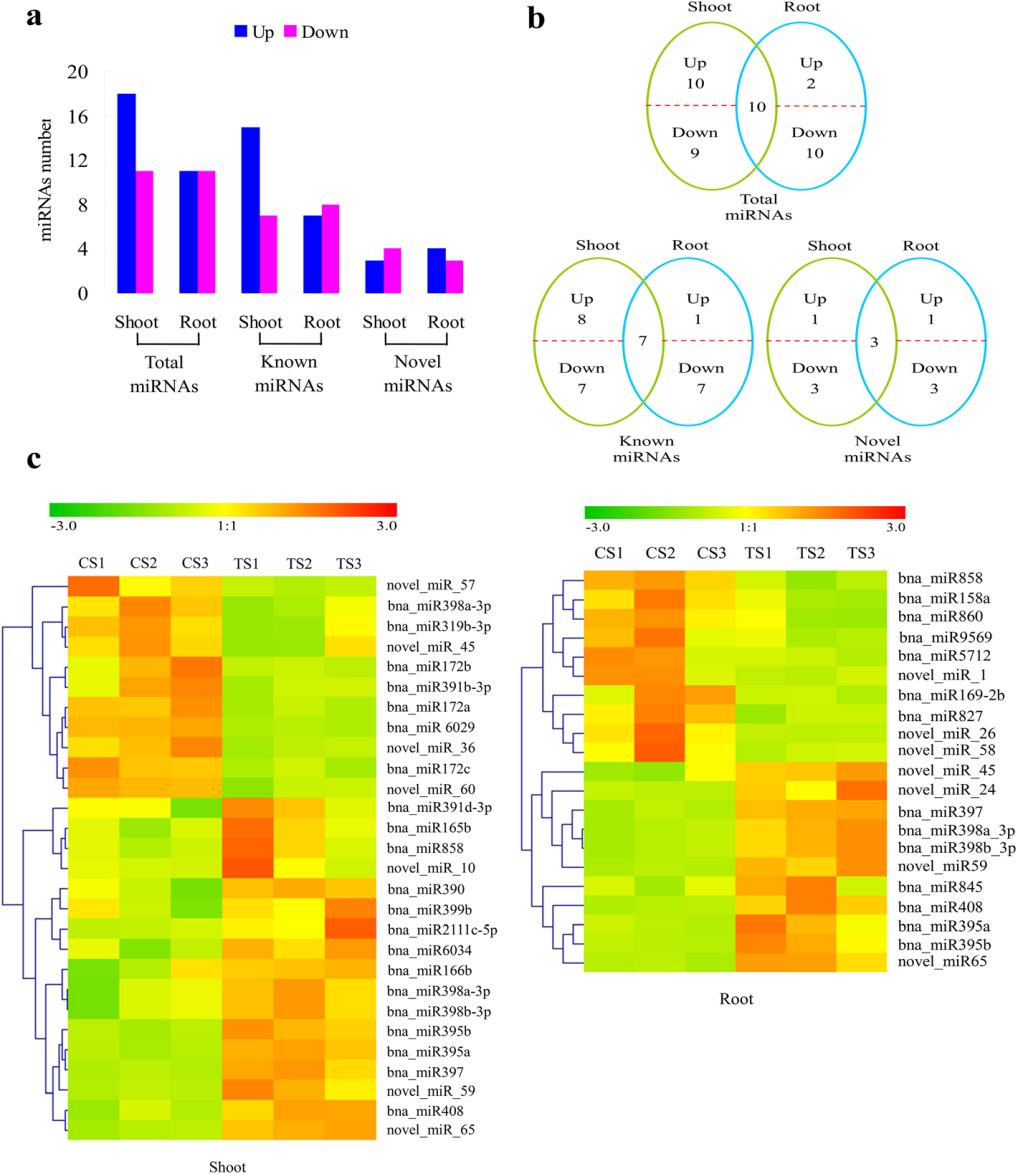
Differentially expressed miRNAs in the root and shoot of *Brassica napus* seedlings in response to cadmium stress. **a** The number of miRNAs up- or down-regulated by Cd treatment by > 2-fold in the root and shoot (*P* < 0.05); **b** A Venn diagrams showing the unique and shared differentially expressed miRNAs in the root and shoot under Cd stress; **c** Hierarchical cluster analysis of differentially expressed miRNAs in the root and shoot. CS1, CS2, CS3 indicate control samples for the shoot, TS1, TS2 and TS3 indicate treatment samples for the shoot, CR1, CR2 and CR3 indicate control samples for the root and TR1, TR2 and TR3 indicate treatment samples for the root. The fold-change ratios of the miRNAs are indicated by different colors

Of the 171 miRNAs, 22 miRNAs in the root and 29 miRNAs in the shoot (20%) were identified as differentially expressed under Cd stress relative to the control (Fig. 3a). Of these differentially expressed miRNAs, ten (seven previously known and three novel) were differentially expressed in both the root and shoot (Fig. 3b), with eight out of ten miRNAs showing up-regulation under Cd stress in both tissues. Expression trends for these miRNAs are shown in Fig. 3c: most differentially expressed miRNAs were up-regulated after Cd treatment in the shoot, while the up- and down-regulated miRNAs showed no significant difference in expression in the root after Cd treatment.

A total of 20 miRNA families were identified in these differentially expressed miRNAs, including 11 families differentially expressed in the root (13 miRNAs) and 14 families differentially expressed in the shoot (20 miRNAs) (Table 1). Of these, five families (MIR395, MIR397, MIR398, MIR408 and MIR858) and three novel miRNAs were differentially expressed in both tissues. All were up-regulated except for one down-regulated miRNA. These miRNAs also displayed the most significant expression changes in response to Cd. The remaining six miRNA families were differentially expressed only in the root (one family up-regulated and five families down-regulated), while nine miRNA families were differentially expressed only in the shoot (five families up-regulated, three families down-regulated, and one miRNA family with both up- and down- regulated miRNAs).

**Table 1:** Significantly differentially expressed miRNA families and miRNAs (*P* < 0.05) in tissues of root and shoot of *Brassica napus* seedlings under Cd stress

Name	Root				Shoot			
	miRNA num.	Up	Down	Mean [log_2_(Ratio)]	miRNA num.	Up	Down	Mean [log_2_(Ratio)]
MIR395 family	2	2	0	2.51	2	2	0	2.38
MIR397 family	1	1	0	2.85	1	1	0	3.84
MIR398 family	2	2	0	2.68	2	2	0	1.07
MIR408 family	1	1	0	3.76	1	1	0	2.03
MIR858 family	1	0	1	1.3	1	1	0	1.8
novel_miR_121	1	1	0	0.61	1	0	1	0.7
novel_miR_164	1	1	0	3.29	1	1	0	3.52
novel_miR_178	1	1	0	3.37	1	1	0	2.3
MIR158 family	1	0	1	0.85	–	–	–	–
MIR169_2 family	1	0	1	1.92	–	–	–	–
MIR827_2 family	1	0	1	0.96	–	–	–	–
MIR845_1 family	1	1	0	0.66	–	–	–	–
MIR860 family	1	0	1	1.45	–	–	–	–
MIR1511 family	1	0	1	1.21	–	–	–	–
novel_miR_160	1	0	1	2.35	–	–	–	–
novel_miR_19	1	0	1	1.07	–	–	–	–
novel_miR_4	1	0	1	1.02	–	–	–	–
novel_miR_71	1	0	1	1.49	–	–	–	–
novel_miR_78	1	1	0	3.86	–	–	–	–
novel_miR_83	1	0	1	1.97	–	–	–	–
MIR166 family	–	–	–	–	2	2	0	1.07
MIR172 family	–	–	–	–	3	0	3	0.77
MIR319 family	–	–	–	–	2	1	1	1.11
MIR390 family	–	–	–	–	1	1	0	1.07
MIR391 family	–	–	–	–	1	0	1	0.72
MIR396 family	–	–	–	–	1	0	1	0.92
MIR399 family	–	–	–	–	1	1	0	0.63
MIR1140 family	–	–	–	–	1	1	0	0.59
MIR2111 family	–	–	–	–	1	1	0	2.1
bna-miR6029	–	–	–	–	1	0	1	0.69
bna-miR6034	–	–	–	–	1	1	0	1.15
novel_miR_103	–	–	–	–	1	0	1	0.77
novel_miR_159	–	–	–	–	1	0	1	0.6
novel_miR_167	–	–	–	–	1	0	1	0.74
novel_miR_38	–	–	–	–	1	1	0	2.01
Total	22	11	11	–	29	18	11	–

### Changes in gene transcript levels are involved in the response to cd in oilseed rape

A total of 44–64 M clean reads were generated from the mRNA expression profiles of 12 mRNA RNA libraries, with 61–70% of reads mapping to the reference genome (Additional file 1: Table S4). In all, 103,816 unigenes consisting of 2776 new genes and 101,040 reference genes were obtained, of which 98.6% (102392) could be annotated.

**Fig. 4:**
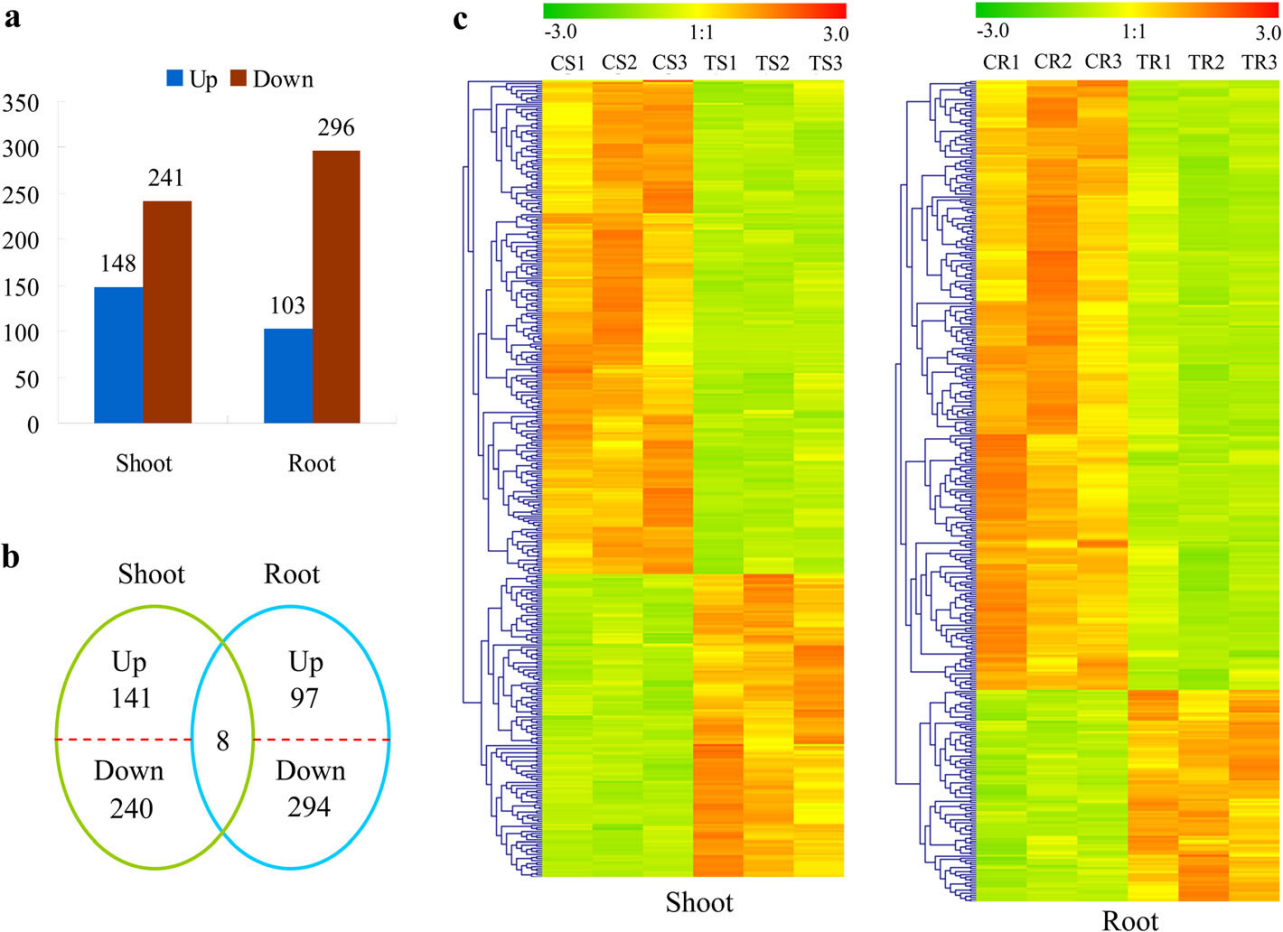
Differentially expressed genes in the root and shoot of *Brassica napus* seedlings in response to cadmium stress. **a** The number of genes up- or down-regulated by Cd treatment by > 2-fold in root and shoot (*P* < 0.05); **b** A Venn diagrams showing the unique and shared differentially expressed genes in the root and shoot under Cd stress; **c** Hierarchical cluster analysis of differentially expressed genes in the root and shoot. CS1, CS2, CS3 indicate control samples for the shoot, TS1, TS2 and TS3 indicate treatment samples for the shoot, CR1, CR2 and CR3 indicate control samples for the root and TR1, TR2 and TR3 indicate treatment samples for the root. The fold-change ratios of the genes are indicated by different colors

Overall, 103 up-regulated and 296 down-regulated genes in the root, 148 up-regulated and 241 down-regulated genes in the shoot under Cd treatment were identified upon Cd treatment (Fig. 4a), of which eight genes were differentially expressed in both root and shoot tissues (Fig. 4b). Except for one differentially expressed gene (DEG) which was up-regulated in the shoot but down-regulated in the root, the remaining seven DEGs showed the same expression patterns in both root and shoot, with six genes up-regulated and one gene down-regulated. Expression patterns of these mRNAs are shown in Fig. 4c: most differentially expressed mRNAs were down-regulated after Cd treatment in both root and shoot tissues.

### Gene ontology (GO) and pathway enrichment revealed the predominance of secondary metabolism, metal ion transport, and antioxidants during the cd response in oilseed rape

In the root, 67, 38 and nine GO terms were significantly enriched for biological processes, molecular function and cellular component annotations (Additional file 1: Table S5). In the shoot, 52, 33 and four GO terms were significantly enriched for biological processes, molecular function and cellular component annotations (Additional file 1: Table S5). Biological processes related to secondary metabolic processes were predominantly enriched in both root and shoot tissues, while processes related to transition metal ion transport and camalexin biosynthesis were especially predominant in the root, and processes related to glucosinolate and indoleacetic acid biosynthesis were especially predominant in the shoot (Additional file 1: Table S5). Molecular functions related to superoxide dismutase were especially predominant in the root, while molecular functions related to transcription factors were especially predominant in the shoot (Additional file 1: Table S5). Cellular components of cell wall and extracellular regions were predominantly enriched in both root and shoot tissues (Additional file 1: Table S5).

**Fig. 5:**
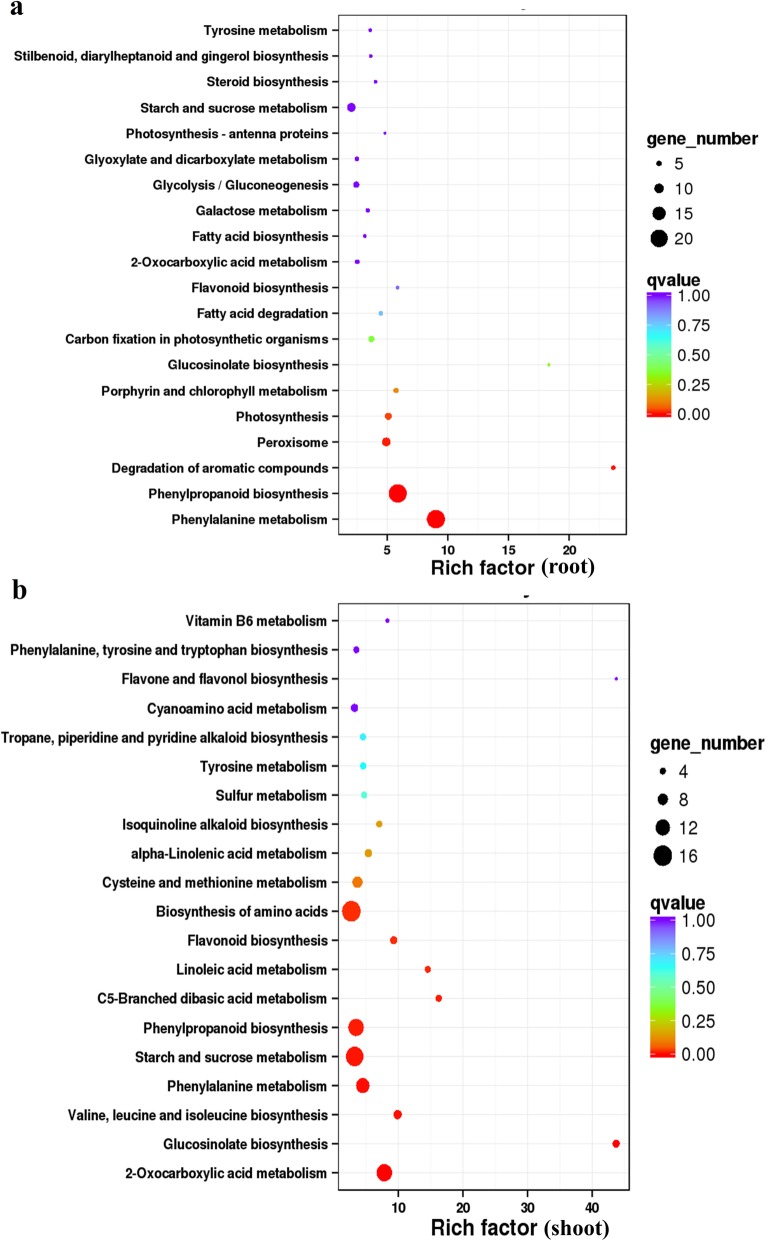
Kyoto Encyclopedia of Genes and Genomes (KEGG) enrichment analysis of differentially expressed genes (DEGs) in the root and shoot of *Brassica napus* seedlings in response to cadmium stress. **a** and **b** represented the top 20 enriched pathways in the root and shoot, respectively. Each circle represents a KEGG pathway, the Y-axis represents the pathway name, and the X-axis represents the enrichment factor, which compares the ratio of genes annotated to a pathway among the DEGs to the ratio of genes annotated to that pathway among all genes. The larger the enrichment factor, the more significant the enrichment of DEGs in the pathway

Pathway enrichment analysis revealed a few important pathways that were significantly enriched in response to Cd stress (Fig. 5). Pathways related to secondary metabolism processes, such as phenylalanine metabolism and in particular phenylpropanoid biosynthesis were the most significantly enriched pathways in the root tissue (Fig. 5). Pathways involved in amino acid biosynthesis, starch and sucrose metabolism, and other similar secondary metabolism pathways, such as the 2-oxocarboxylic acid metabolism, phenylalanine metabolism, phenylpropanoid biosynthesis were the most significantly enriched pathways in the shoot tissue.

### DEGs related to key biological processes

#### DEGs involved in cd transport

Metal transporters have been revealed to mediate heavy metal uptake and translocation in plants [36]. In the present study, 18 and 12 known heavy metal transporters were identified in the root and shoot, including one ZIP (ZRT/IRT-like Proteins), three YSL (yellow Stripe 1-like transporters), four PPase (pyrophosphatase), six NRT (nitrate transporters), two COPT (copper transport protein family), three CML (calcium-binding proteins), two CAM (calmodulin), five ABC (ATP Binding Cassette) transporters, one CAX (vacuolar cation/proton exchanger), and one ATP synthase gene (Table 2, Additional file 1: Table S6). DEGs encoding YSL, PPase, NRT, and ABC transporters were common to both the root and shoot, while DEGs encoding ZIP, COPT, CML and CAM were specific to the root, and DEGs encoding CAX and ATP synthase were specific to the shoot. All genes encoding ZIPs, YSL, COPT, CAM, ABC transporters, and CAX tended to be up-regulated, while all genes encoding PPase, NRT, and ATP synthase were down-regulated.

**Table 2:** Differentially expressed genes related to cadmium (Cd) transport, transcriptional regulation, and secondary metabolism in roots and shoots of oilseed rape seedlings in response to Cd treatment

Types	root			shoot		
	total	up	down	total	up	down
Cd transport	18	11	7	12	7	5
Transcription regulation	22	6	16	46	17	29
Secondary metabolism	53	6	47	58	17	41
Antioxidant response	58	9	49	46	24	22
Chlorophyll synthesis and photosynthesis	16	0	16	9	0	9

#### DEGs involved in regulation of transcription

Of the 780 genes differentially expressed in the root and shoot of oilseed rape seedlings in response to Cd, 67 genes (8.6%) were involved in transcriptional regulation (Additional file 1: Table S7). Interestingly, more than twice as many differentially expressed transcription factors (TFs) were found in the shoot (46 DEGs belonging to 18 TF families) than in the root (22 DEGs belonging to seven TF families) (Table 2, Additional file 1: Table S7). Five TF families were differentially expressed in both shoot and root (Additional file 1: Table S7). Of the differentially expressed TFs, more were down-regulated (29 and 16 in root and shoot respectively) than up-regulated (17 and six in root and shoot respectively) (Table 2, Additional file 1: Table S7).

#### DEGs involved in secondary metabolic processes

A total of 53 DEGs in the root and 58 DEGs in the shoot were found to be involved in secondary metabolic processes (Table 2, Additional file 1: Table S8). These were involved in eight secondary metabolic pathways: glucosinolate biosynthesis and catabolism, lignin biosynthesis and catabolism, phenylpropanoid metabolism, toxin catabolism, unsaturated fatty acid biosynthesis, flavonoid/flavonol/anthocyanin biosynthesis, and coumarin biosynthesis. DEGs involved in lignin biosynthesis and cutin biosynthesis were especially prominent in the shoot and root, respectively (Table 2). The highest numbers of DEGs involved in secondary metabolism processes were related to glucosinolates in both the root (17 DEGs; 14 involved in biosynthesis and three involved in catabolism) and shoot (37 DEGs; 36 involved in biosynthesis and one in catabolism) (Table 2). All 14 DEGs in the root and 33/36 of the DEGs in the shoot involved in glucosinolate biosynthesis were down-regulated (Table 2). Similarly, DEGs related to lignin biosynthesis, phenylpropanoid metabolism, toxin catabolism, and coumarin biosynthesis also showed a trend of down-regulation in both root and shoot (Table 2). By contrast, DEGs related to lignan biosynthesis, unsaturated fatty acid biosynthesis and flavonoid/flavonol/anthocyanin biosynthesis tended to be down-regulated in the root, but up-regulated in the shoot (Table 2).

#### DEGs involved in antioxidant response

A total of 58 DEGs in the root and 46 DEGs in the shoot encoded different antioxidant families (Table 2, Additional file 1: Table S9). Half of the DEGs (52%) in the shoot were up-regulated, while most DEGs (85%) in the root were down-regulated. The antioxidant families peroxidase (POD), 2-oxoglutarate (2-OG) dioxygenase, laccase, and monooxygenase (MO) were induced in both the root and shoot, but the expression trend was opposite between the two tissues, with most DEGs down-regulated in the root but up-regulated in the shoot. Many of the antioxidant families were specific between tissues for Cd response, such as the copper/zinc superoxide dismutase (SOD), Fe-SOD, ferretin, NAD(P)-binding Rossmann-fold protein, and zinc-binding dehydrogenase (ZBDH) families, which were specifically induced in the root, and the lipoxygenase, copper amine oxidase (CuAO), ferredoxin:NADP(H) oxidoreductase (FNR), and nitrite reductase (NIR) families which were specifically induced in the shoot. These findings suggested that antioxidant-encoding genes may play important roles in reducing Cd toxicity in both root and shoot in *B. napus*, but that the mechanism of protection may be distinct between tissues.

#### DEGs involved in photosynthesis

We identified 25 DEGs related to photosynthesis. All of these DEGs were down-regulated in response to Cd treatment (Table 2, Additional file 1: Table S10). These genes were mainly involved in photosystem subunit-related processes, chlorophyll-protein complex subunit encoding, rubisco small subunit encoding, electron transport, carbon reduction, and other photosynthesis processes.

### A close crosstalk was observed between several cd induced miRNAs and mRNAs

miRNA-mRNA interaction pair means the anti-regulation of a miRNA and a corresponding mRNA. A total of eight anti-regulation miRNA-mRNA interaction pairs, involving three miRNA families and six target mRNAs, were identified in the root (Table 3, Additional file 1: Table S11). This included seven pairs with up-regulated miRNAs and down-regulated mRNAs, and only one pair with down-regulated miRNA and up-regulated mRNA (Table 3, Additional file 1: Table S11). Of the targets, one down-regulated target of miR395 comprised an ABA-binding protein encoding magnesium chelatase, which is involved in plastid-to-nucleus signal transduction and is related to chlorophyll biosynthetic process and photosynthesis. The four targets of miR398, two encoding chloroplastic copper/zinc superoxide dismutase 2 (CSD2), one encoding cytosolic copper/zinc superoxide dismutase (CSD1), and one encoding copper-zinc superoxide dismutase copper chaperone (CCS), were also down-regulated,. Thus, the four targets of miR398 were highly correlated and all were involved in the CCS-dependent detoxification of superoxide radicals pathway, which is regulated.

**Table 3:** The anti-regulation miRNA-mRNA interaction pairs in root and shoot tissues of *Brassica napus* seedlings in response to Cd stress

Tissue	miRNA Family	miRNA Name	Targets	miRNA expression level			mRNA expression level			Target annotation
				log_2_Ratio	FDR	regulate	log_2_Ratio	FDR	regulate	
root	MIR395	bna-miR395a	BnaA03g04440D	3.01	0.0000	up	−3.22	0.0012	down	ABA-binding protein (ABAR)
root	MIR398	bna-miR398a-3p	BnaA04g16310D	2.85	0.0000	up	−1.69	0.0011	down	chloroplastic copper/zinc superoxide dismutase 2 (CSD2)
root	MIR398	bna-miR398b-3p	BnaA04g16310D	2.32	0.0000	up	−1.69	0.0011	down	
root	MIR169_2	bna-miR169_2b	BnaC03g08060D	−1.92	0.0076	down	4.74	0.0034	up	NAD(P)H dehydrogenase B4 protein (NDB4)
root	MIR398	bna-miR398a-3p	BnaC04g39580D	2.85	0.0000	up	−1.77	0.0000	down	chloroplastic copper/zinc superoxide dismutase 2 (CSD2)
root	MIR398	bna-miR398b-3p	BnaC04g39580D	2.32	0.0000	up	−1.77	0.0000	down	
root	MIR398	bna-miR398b-3p	BnaC08g42970D	2.32	0.0000	up	−2.82	0.0015	down	cytosolic copper/zinc superoxide dismutase (CSD1)
root	MIR398	bna-miR398b-3p	BnaCnng33420D	2.32	0.0000	up	−1.85	0.0000	down	copper-zinc superoxide dismutase copper chaperone (CCS)
shoot	MIR2111	bna-miR2111c-5p	BnaA09g44060D	2.1	0.1599	up	−2	0.0001	down	1-aminocyclopropane-1-carboxylate oxidase (ACO1)
shoot	MIR395	bna-miR395a	BnaC09g46440D	2.83	0.0000	up	−2.01	0.0000	down	sulfate transporter 2;1 (SULTR2;1)
shoot	MIR395	bna-miR395b	BnaC09g46440D	2.83	0.0000	up	−2.01	0.0000	down	
shoot	MIR397	bna-miR397	BnaC04g07220D	3.84	0.0000	up	−2.29	0.0041	down	laccase-like multicopper oxidase 4 (LAC4)
shoot	MIR397	bna-miR397	BnaA07g26900D	3.84	0.0000	up	−3.22	0.0001	down	zinc finger C-×8-C-×5-C-×3-H type family protein (CDM1)
shoot	MIR408	bna-miR408	BnaA04g11970D	2.03	0.0001	up	−1.36	0.0032	down	proline-rich protein (PRP2)
shoot	MIR398	bna-miR398b-3p	BnaA02g18700D	1.07	0.0530	up	−1.29	0.0007	down	FCS like zinc finger 6 (FLZ6)
shoot	NA	bna-miR6029	BnaA03g11200D	−0.65	0.1559	down	1.01	0.0035	up	inner centromere protein (INCENP)

by biotic and abiotic stress. In the shoot, eight anti-regulation miRNA-mRNA interaction pairs, involving five miRNA families, one novel miRNA, and seven target mRNAs, were identified (Table 3). Of these, seven pairs were up-regulated miRNAs and down-regulated mRNAs, only one pair displayed down-regulated miRNA and up-regulated mRNA (Table 3). The seven target mRNAs were mainly involved in oxidation-reduction processes and plant organ development.

### The expression profile data was validated by real-time RT-PCR

To validate the expression data, the relative expression levels of 15 randomly selected differentially expressed mRNAs and ten miRNAs from our sequencing data were investigated with qRT-PCR. Our results showed that the qRT-PCR analysis for 13 of the 15 differentially expressed mRNAs and seven of the ten differentially expressed miRNAs displayed similar expression patterns to those generated from high-throughput sequencing (Additional file 2: Figure S1), thus confirming the reliability of the data provided by RNA-Seq.

## Discussion

### miRNA regulation of genes in response to cd stress in oilseed rape

Co-analysis of the expression of miRNA and their putative mRNA targets in response to Cd stress revealed 7/8 anti-regulation pairs in the root (three miRNA families targeting six mRNAs) and 7/8 anti-regulation pairs in the shoot (five miRNA families targeting seven mRNAs) where miRNA up-regulation was coupled with down-regulation of the mRNA, with the remaining two pairs showing miRNA down-regulation coupled with mRNA up-regulation. Several of these targeted genes were involved in processes expected to be initiated in response to Cd stress. In the shoot, four related genes (an A4 and a C4 copy of CSD2, a CSD1 and a CCS gene) targeted by a single miRNA, belong to the copper/zinc superoxide dismutase genes, which were involved in the CCS-dependent detoxification of superoxide radicals pathway, and were regulated by biotic and abiotic stress [37, 38], while another gene in the root was also involved in oxidation-reduction processes and defense response (an *ACO1* gene homolog). Other genes were putatively involved in chlorophyll biosynthesis/photosynthesis pathways, sulfate transport, cell wall biosynthesis and organization, cell growth and transcriptional regulation, similar to the categories which we also found to be enriched for differentially expressed genes in response to Cd stress. Our genome-wide study highlights both miRNAs and genes which would make excellent targets for detailed experimental investigation of Cd response in oilseed rape in future.

### The expression of a range of miRNAs were changed under cd stress in oilseed rape

Zhou et al. (2012) previously identified eight miRNA families differentially expressed in response to Cd stress in *B. napus* roots, and 13 miRNA families differentially expressed in response to Cd stress in *B. napus* shoots [35]. In the present study, a total of 11 and 14 miRNA families were identified to be responsive to Cd stress in roots and shoots, respectively (Table 1). As expected, several of the Cd-responsive miRNAs previously identified by Zhou et al. (2012) also showed differential regulation under Cd stress in our study [35]. For instance, miR319, miR395, miR398, and miR2111, were up-regulated under Cd stress, miR158 and miR172 were down-regulated, whereas miR585 were down-regulated in root and up-regulated in shoot. However, the miR161, miR164, miR171, miR319, miR394, miR400, miR857 and miR1885 families detected in Zhou et al. 2012 were not significantly differentially regulated under Cd stress in our study, while the miR166, miR169, miR390, miR391, miR396, miR397, miR399, miR408, miR818, miR827, miR845, miR858, miR860, miR1140 and miR1511 families detected in our study were not detected by Zhou et al. (2012). These differences might be caused by the *B. napus* genotype used or by the longer period of our Cd treatment relative to this study. Thus, further functional studies are needed to validate the precise regulatory roles of these miRNAs in response to Cd in *B. napus*.

### Cd stress significantly influences the photosynthetic pathway in oilseed rape seedlings

Cd can influence photosynthetic pigments, causing a decrease in chlorophyll content and thus affecting photosynthetic function [39, 40]. In our study, the total chlorophyll content showed a very significant decrease five days after treatment under 5 mg/L, 10 mg/L and 20 mg/L Cd^2+^ (Fig. 1b), and the minimum chlorophyll content occurred after 10 days of treatment when compared with the control. The decrease in chlorophyll content in our study was similar to that observed by Ali et al. (2013b) [41] and Ding et al. (2018) [25], who showed that chlorophyll content significantly decreased with increasing Cd treatment. We found that the chlorophyll decrease occurred only for younger leaves, but did not decrease the chlorophyll content of older leaf (Fig. 1a). However, unlike the results reported by Ali et al. (2013b) [41], which showed that chlorophyll content significantly decreased as Cd concentration increased (0, 100, and 500 μM), we found that higher Cd concentration arrested the development of younger leaves, causing stability and even a gradual increase in chlorophyll content in older leaves after a long time in high Cd concentration treatment.

Chloroplast proteins play important roles in chloroplast biogenesis [42]. Defects in chloroplast proteins such as chloroplast protein import subunit (toc36), ATP synthase subunit (atpD), cytochrome b6-f complex iron–sulfur subunit and chloroplast mRNA maturation enzyme, could cause the chlorophyll-deficient phenotype [43]. In the present study, the expression of several chloroplast proteins, including two rubisco small subunit encoding genes, one rubisco activase gene, and three NAD(P)H dehydrogenase genes, was significantly down-regulated in the shoot tissue of *B. napus* under Cd stress (Additional file 1: Table S10). Izumi et al. (2012) determined that the mutation of two rubisco small subunits yielded a pale green leaf colour in *Arabidopsis thaliana* [44]. Suorsa et al. (2009) reported that the NDH-deficient plants do not show any phenotype related to greening, but affect cyclic electron transfer around photosystem (PS) I during the photosynthesis process [45]. Thus, it is not surprising that two rubisco small subunit encoding genes and one rubisco activase gene were significantly down-regulated under Cd stress; this might be closely related to the chlorophyll-deficiency phenotype, and linked to the down-regulation of several photosynthesis-related genes.

Although roots do not make chlorophyll or photosynthesise, 16 genes involved in plant chlorophyll synthesis or in the photosynthetic process were identified in response to Cd in the root. Thus, despite the gene annotation predictions, these genes must have functions other than photosynthesis. For instance, it has been reported that the overexpression of light-dependent protochlorophyllide oxidoreductase PORA or PORB provides protection against photooxidative damage by overexpression of light-dependent protochlorophyllide oxidoreductase PORA or PORB [46]. As well, the magnesium-chelatase H subunit functions in abscisic acid signaling to regulate plant adaptation to environmental stress challenges, by regulating stomatal aperture and expression of stress-responsive genes [47]. The pyridine nucleotide-disulphide oxidoreductases motifs are highly conserved in the deduced amino acid with sequences from glutathione reductase, which plays an essential role in cell defense against reactive oxygen metabolites [48]. Thus, we speculate that the differential expression of chlorophyll- or photosynthesis-related genes in the roots involves cellular protection mechanisms against the environmental stress caused by Cd.

### Glutathione (GSH) metabolism is involved in cd response in both roots and shoots of oilseed rape seedlings

**Fig. 6:**
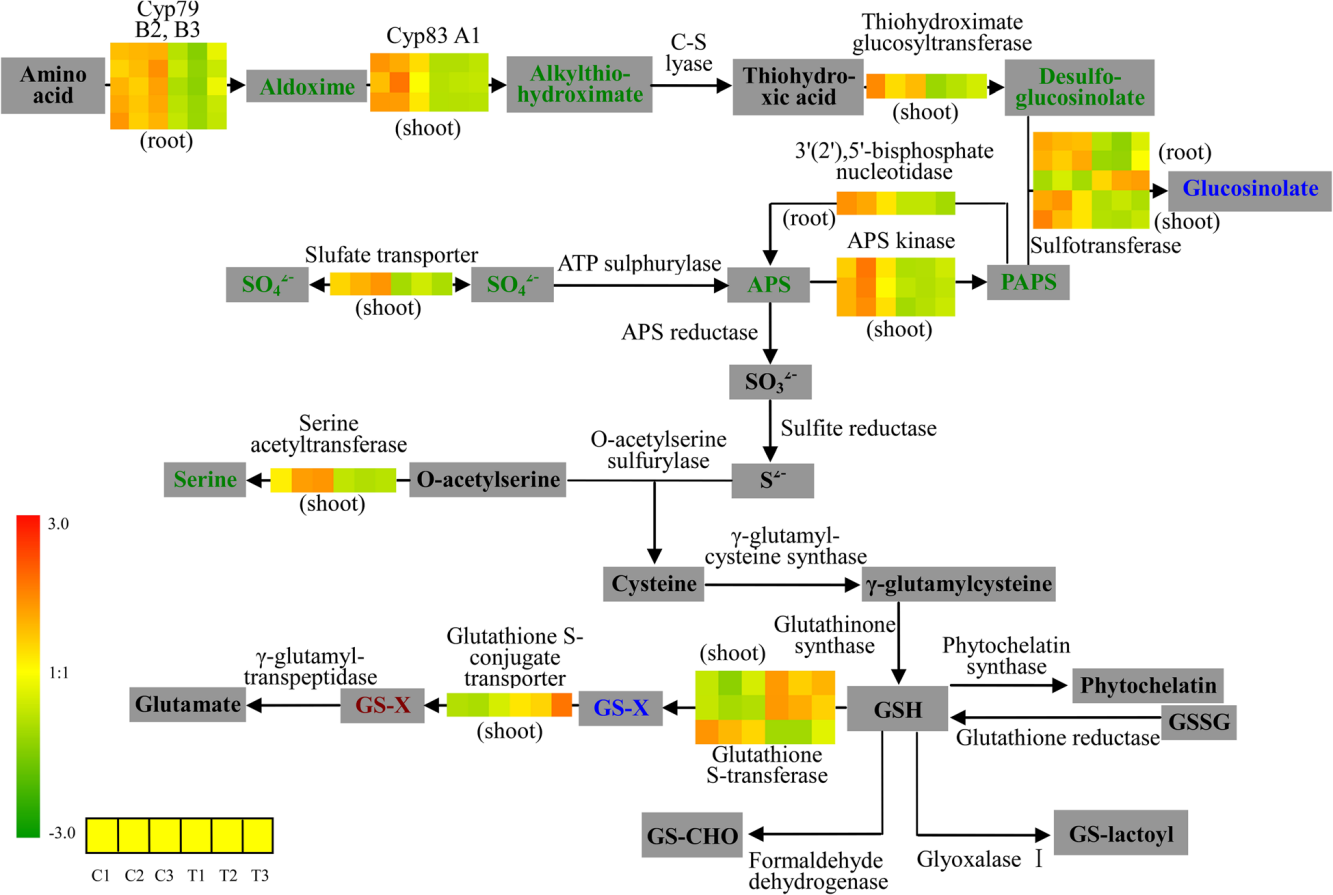
Transcriptional changes of genes involved in sulfur and GSH metabolism in the root and shoot of cadmium-treated *Brassica napus* seedlings. The metabolites are represented in gray boxes, arrows represent enzymatic reactions, and names of the enzymes and tissues are given under or above the colored bars. Colored bars on the arrows indicate relative expression of the corresponding genes across the three different biological replicates for the control (C1, C2 and C3; left) and cadmium treatment (T1, T2 and T3; right). Products with red and green color representations indicate an increase and decrease in products respectively, while the products with represented by blue color indicate products with both up- and down-regulation

We found that four genes closely co-located downstream of tripeptide glutathione (GSH) were significantly differentially expressed in our study. GSH plays a significant role in stress response [49]. When plants respond to heavy metals, GSH is the substrate for biosynthesis of the heavy metal-binding peptides phytochelatins (PCs), which form metal-thiolate bonds and PC-metal complexes and are then sequestered to the vacuoles [50]. In *Arabidopsis thaliana*, GSH-deficient mutants with 15–30% of wild-type levels of GSH show Cd sensitivity [51]. Of the four genes located close together downstream of GSH and significantly differentially expressed in our study (Fig. 6), three were up-regulated only in the shoot, suggesting tissue-specific regulation of gene expression. We failed to observe transcriptional regulation of sulfite reductase gene and downstream genes involved in cysteine and GSH biosyntheses in response to Cd treatment (Fig. 6), despite the fact that most of these genes play a key role in Cd sensitivity (e.g. gamma-glutamylcysteine synthase (GSH1) [12]. Similar results were also found in *Arabidopsis thaliana*: although GSH1 enzyme activity increases rapidly in response to Cd in a dosage-dependent manner [52] and glutamylcysteine levels also increase in response to Cd [53], the expression level of these genes shows no significant change. Our results confirm that sulfite reductase and other downstream genes involved in cysteine and GSH biosynthesis are post-transcriptionally regulated in response to Cd in *B. napus*, as already demonstrated for other stresses by May et al. (1998a) [54]. Also, in our analyses, genes encoding PC synthases showed no expression change after Cd treatment (Fig. 6). In previous studies, PC synthases were found to be constitutively expressed and post-transcriptionally regulated by activation of the enzyme in the presence of the metal [15].

Glutathione S-transferase (GST) and glutathione S-conjugate transporters are a group of multifunctional enzymes which catalyze conjugation of GSH with xenobiotic compounds for plant detoxification. In our analysis, two of three DEGs encoding glutathione S-transferase and one glutathione S- conjugate transporter were up-regulated in the shoot in response to Cd (Fig. 6). Research in yeast cells has found that GST is involved in GSH-Cd formation to decrease Cd uptake [55]. GST also functions as glutathione peroxidase (GPOX) and participates in plant defense against oxidative stress and toxicity generated from heavy metals: Moons et al. (2003) found that Cd, Co, Ni and Zn significantly enhance the expression of GST genes in rice [56]. Thus, the up-regulation of GST and glutathione S-conjugate transporter in Cd response in oilseed rape seedlings suggests similar regulatory mechanisms to those in rice, such that the up-regulation of GST is associated with plant Cd detoxification.

### Phenylpropanoid metabolism was involved in cd response in both roots and shoots of oilseed rape seedlings

**Fig. 7:**
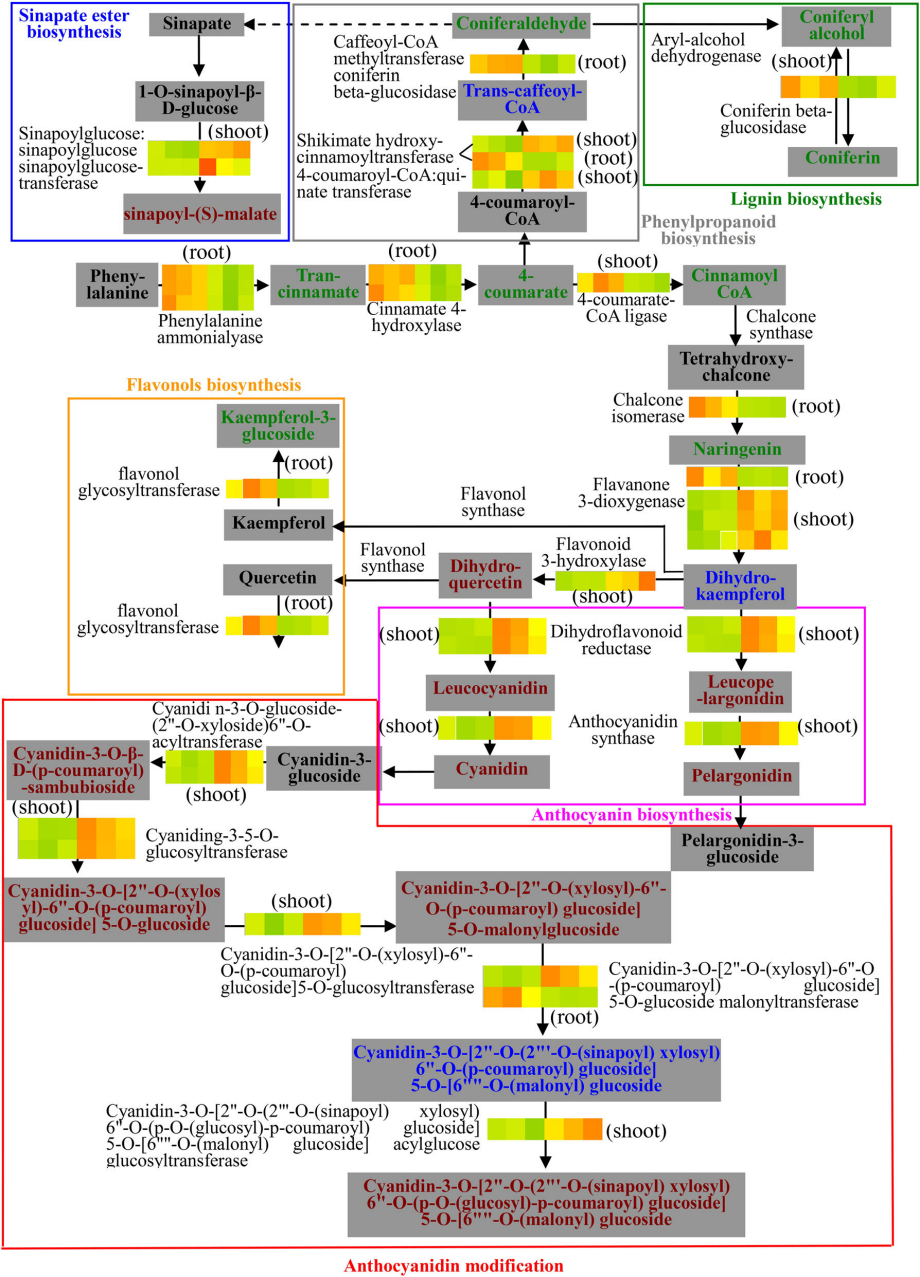
Transcriptional changes of genes involved in phenylpropanoid metabolisms in the root and shoot of cadmium-treated *Brassica napus* seedlings. The metabolites are represented in gray boxes, arrows represent enzymatic reactions, and names of the enzymes and tissue type are given under or above the colored bars. Colored bars on the arrows indicate relative expression of the corresponding genes across the three different biological replicates for the control (C1, C2 and C3; left) and cadmium treatment (T1, T2 and T3; right). Products with red and green color representations indicate an increase and decrease in products respectively, while the products with represented by blue color indicate products with both up- and down-regulation

Cd accumulation in plants causes the accumulation of reactive oxygen species (ROS) [57]. ROS can directly damage a wide range of cellular components, such as mitochondria [58], chloroplasts [59], and microtubules [60]. Enhancing ROS-scavenging is an important plant Cd response mechanism, and phenylpropanoids and their derivatives are well-known ROS scavengers. Several reports have demonstrated that diverse forms of biotic and abiotic stress result in the accumulation of one or more phenolic compounds [61]. In the present study, 12 and 23 DEGs in roots and shoots were directly involved in pathways related to phenylpropanoids and phenylpropanoid derivatives (Fig. 7). In this metabolism pathway, we found all 12 DEGs in the root were down-regulated but that 21/23 DEGs in the shoot were up-regulated, suggesting different regulation of root and shoot tissues. Additionally, most of the DEGs in the shoot were involved in the anthocyanin biosynthesis and modification pathways, but only one gene in these two pathways was found in the roots, suggesting that while the phenylpropanoid-related Cd response for the shoot was mostly anthocyanin-related, anthocyanins in the root are not involved. These findings in oilseed rape are similar in part to those of Herbette et al. (2006), who demonstrated that genes involved in phenylpropanoid and anthocyanin production are differentially expressed in *Arabidopsis thaliana* exposed to Cd treatment [52]. However, Herbette et al. (2006) found up-regulated expression of genes involved in phenylpropanoid and anthocyanin production only in leaves of Cd-treated plants, in contrast to our data [52]. This difference may correspond to transitory effects at different time points under Cd stress.

### Antioxidant response was a crucial cd-induced response in both roots and shoots of oilseed rape seedlings

The oxidative stress induced by Cd has been shown to play an important role in plant toxicity [62]. Under normal conditions, the level of reactive oxygen species (ROS) remains low and does not pose a hazard to plants. However, ROS metabolism is blocked after Cd stress, and excess ROS, such as superoxide anions, hydroxyl ions, and hydrogen peroxide, accumulate in the cell, cause membrane lipid peroxidation, disrupt the electron transport chain, and lead to oxidative damage in plants [63]. Several studies have found that proline and malondialdehyde content were increased after Cd stress, and that antioxidants such as peroxidase, superoxide dismutase, hydrogen peroxidase, GSH and GST also increased [64], suggesting the stimulation of the antioxidant system. In agreement with the above physiological results, our data found that a set of genes encoding antioxidants such as POD, SOD, Fe-SOD, FNR and lipoxygenase were induced by Cd stress. However, opposite expression trends and tissue-specific antioxidant families were detected between the tissues of the root and shoot, which suggests that the Cd-induced stress response was distinct between tissues, as also found by Zhou et al. (2012) [28] in *B. napus* and Herbette et al. (2006) in *A. thaliana* [52].

### TFs play a crucial role in cd response in both roots and shoots of oilseed rape seedlings

TFs are known to play crucial roles in response to abiotic stresses in plants. In our study, the majority of differentially expressed TFs belonged to the TF families (bHLH) DNA-binding proteins, bZIP, zinc finger, ERF and MYB, and WRKY. In *Arabidopsis thaliana* and soybean, bHLH transcription factors like bHLH38, bHLH39, bHLH29 and ORG3-Like have been identified to enhance Cd tolerance via decreased cadmium transfer from roots to shoots, and to improve the iron homeostasis and concentration of shoots [65–67]. In *Arabidopsis thaliana*, the zinc-finger protein AtZAT6 showed significant correlation with Cd tolerance [65], while the TF families of WRKY, bZIP, ERF, and MYB, are associated with Cd stress in species such as tobacco, maize, walnut and creep bentgrass [68–71]. We also observed several other TF families expressed in response to Cd treatment (Table 2). Briefly, the evidence for direct or indirect roles of TFs in response to Cd is quite limited. Our study may provide some suggestions regarding the mechanisms of Cd tolerance at the transcriptional level for future research.

## Conclusions

Cadmium stress induced concurrent expression changes of miRNAs and mRNAs in *B. napus* seedlings. The shoots and roots showed several differentially expressed miRNAs in common, but there was very little overlap between tissue types for differentially expressed mRNAs in response to Cd. Several anti-regulation miRNA-mRNA interaction pairs were identified in response to Cd, and involved key plant stress response pathways such as detoxification of superoxide radicals. The photosynthetic pathway, transcription factor activation, antioxidant response and secondary metabolic processes were identified as major components of response to cadmium in *B. napus* seedlings. Our results identify key genes and processes for further investigation of Cd stress responses in *B. napus*.

## Methods

### Plant materials, cd treatment and chlorophyll content measurements

Seeds of *B. napus* cultivar ‘Zheyou 29’ were collected from the Institute of Crop and Nuclear Technology Utilization, Zhejiang Academy of Agricultural Sciences, Hangzhou, China, and used in this study. No other permissions were necessary to collect samples. Seeds of ‘Zheyou 29’were surface-sterilized and germinated on Petri dishes with deionized water at 25 °C for three days in the dark. Germinated seeds were grown in plastic pots in growth chambers under 14 h light (25 °C) and 10 h dark (18 °C) conditions for three weeks. Seedlings were transplanted into plastic containers containing half-strength Hoagland nutrient solution for one week. 28-day-old seedlings were exposed to treatment with CdCl_2_ at six concentrations: 0 mg/L (control), 5 mg/L, 10 mg/L, 20 mg/L, 30 mg/L, and 40 mg/L of Cd^2+^. New unfolding leaves were measured for total chlorophyll content according to the methods reported in Yin et al. (2008) [72]. About 0.1 g of leaf tissue was homogenized in 5 mL of mixture with acetone: ethanol: water at a 9: 9: 2 (v / v / v) ratio, incubated in the dark for 8–12 h, and then centrifuged at 3500 r/min for 10 min. The absorbance of the supernatant was then measured at 652 nm. Eight measurements of chlorophyll content were taken at two-day intervals. Based on these results maximum decreases in chlorophyll content occurred at 10 days post-treatment under 10 mg/L Cd^2+^ concentration, so the roots and shoots under this condition were then collected separately for high-throughput sequencing and qRT-PCR. The collected samples were frozen in liquid nitrogen immediately and stored at − 80 °C.

Total RNA was extracted from roots and shoots of oilseed rape seedlings after 10 days of 10 mg/L Cd^2+^ treatment and for the control treatment using EZ-10 RNA Miniprep Kits (Sangon Biotech Co., Ltd., Shanghai, China), with three biological replicates for each sample. RNA purity was checked using the NanoPhotometer spectrophotometer (IMPLEN, CA, USA). RNA concentration was measured using a Qubit RNA Assay Kit in a Qubit 2.0 Fluorometer (Life Technologies, CA, USA). RNA integrity was assessed using the RNA Nano 6000 Assay Kit of the Agilent Bioanalyzer 2100 system (Agilent Technologies, CA, USA).

### Small RNA and transcriptome sequencing

A total amount of 2.5 ng RNA per sample was used as input material for the RNA sample preparations. We constructed 12 small RNA sequencing libraries: root control, root treatment under 10 mg/L Cd2+ concentration, shoot control, and shoot treatment under 10 mg/L Cd2+ concentration, with three biological replicates for each sample, using NEBNext®Ultra™ small RNA Sample Library Prep Kit for Illumina® (NEB, USA) following manufacturer’s recommendations. Briefly, total RNA quantity and purity were assayed with an Agilent 2100 Bioanalyzer (Agilent Technologies, Palo Alto, CA) to ensure quality. Then, sRNAs of 18–30 nt were purified from 10 μg of total RNA by 15% polyacrylamide gel electrophoresis. Subsequently, 5′ and 3′ RNA adapters were ligated on both ends using T4 RNA ligase. The adapter-ligated sRNAs were transcribed to cDNA using Super-Script II Reverse Transcriptase and PCR-amplified. Finally, the PCR products were purified and sequenced on an Illumina HiSeq X Ten platform by Biomarker Technologies Co., Ltd. (Beijing, China) and single-end reads were generated.

A total amount of 1 μg RNA per sample was used as input material for the RNA sample preparations. 12 transcriptome sequencing libraries: root control, root treatment under 10 mg/L Cd2+ concentration, shoot control, shoot treatment under 10 mg/L Cd2+ concentration, with three biological replicates for each sample, were generated using NEBNext UltraTM RNA Library Prep Kit for Illumina (NEB, USA) following manufacturer’s recommendations. mRNA with Poly (A) was purified from the total RNA, using oligo(dT) magnetic beads, and then fragmented with an RNA fragmentation kit. First strand cDNA was synthesized using random hexamer primer and M-MuLV Reverse Transcriptase (RNase H-). Second strand cDNA synthesis was subsequently performed using DNA Polymerase I and RNase H. In order to preferentially select cDNA fragments of 200–250 bp in length, the library fragments were purified with AMPure XP system (Beckman Coulter, Beverly, USA). Then, 3 μl USER Enzyme (NEB, USA) was used with size-selected, adaptor-ligated cDNA at 37 °C for 15 min followed by 5 min at 95 °C before PCR. Then PCR was performed with Phusion High-Fidelity DNA polymerase, Universal PCR primers and Index (X) Primer. At last, PCR products were purified (AMPure XP system) and library quality was assessed on the Agilent Bioanalyzer 2100 system. The library preparations were sequenced on an Illumina Hiseq 2500 platform by Biomarker Technologies Co., Ltd. (Beijing, China), for RNA sequencing, and paired-end reads were generated.

### Sequencing data analysis

For small RNA sequencing data, clean reads were firstly obtained by removing contaminants, adaptors, low-quality reads, and reads either shorter than 15 nt or longer than 30 nt. The remaining unique RNAs were mapped to the *B. napus* reference genome (http://www.genoscope.cns.fr/brassicanapus/) using the SOAP2 program [73]. Sequences with a perfect match were retained for further analysis. Using Bowtie tools “soft”, the clean reads were aligned respectively with the Silva database (http://www.arb-silva.de/), the GtRNAdb database (http://lowelab.ucsc.edu/GtRNAdb/), the Rfam database (http://rfam.xfam.org/), and the Repbase database (http://www.girinst.org/repbase/) in order to filter out ribosomal RNA (rRNA), transfer RNA (tRNA), small nuclear RNA (snRNA), small nucleolar RNA (snoRNA) and other ncRNA and repeats. The remaining reads were used to detect known miRNA and novel miRNA predicted by comparison to the genome reference and with known miRNAs from miRBase (http://www.mirbase.org/index.shtml). Mireap software (https://sourceforge.net/projects/mireap/) was used for novel miRNA secondary structure prediction. The miRNA expression was normalized using the formula: normalized expression = actual miRNA count/total count of clean reads × 1,000,000. The fold change was calculated as: fold change = log_2_(Ratio). |log2(FC)| ≥ 0.59 was used to determine the differentially expressed miRNAs. Differential expression analysis was performed using the DESeq R package (1.10.1). DESeq provides statistical routines for determining differential expression in digital miRNA expression data using a model based on the negative binomial distribution. The resulting *P* values were adjusted using the Benjamini and Hochberg’s approach for controlling the false discovery rate. Fold change, *P* values and false discovery rate (FDR) values were used together to screen for differentially expressed miRNAs. The website psRNATarget 2011 (http://plantgrn.noble.org/psRNATarget/) was used to predict target genes of miRNAs according to default parameters.

For transcriptome data, clean reads were screened from raw data by removing contaminants, adaptors, and low-quality reads. The software “TopHat2” was used to map reads to the reference genome (*Brassica napus* “Darmor-bzh” v4.1). Gene expression quantification was estimated by fragments per kilobase of transcript per million fragments mapped. The DEGseq (2010) R package was used to analyze the differentially expressed genes (DEGs) with a FDR < 0.001 and |log_2_(Ratio)| ≥ 1. The GOseq R package based on Wallenius non-central hyper-geometric distribution was used to perform the GO enrichment analysis of the target genes [74]. We used the whole genome as the default background distribution. Resampling was performed by randomly selecting a set of genes the same size as the set of DEGs, and counting the number of genes associated with the GO category of interest. The resampling was repeated many times and the resulting distribution of GO category membership was taken to approximate the shape of the true probability distribution. This sampling distribution allowed calculation of a *P*-value for each GO category being over-represented in the set of differentially expressed genes. *P-*values less than 0.05 and a number of DEGs in each GO category exceeding the expected gene number associated with the GO category were used to identify over-representation of a GO category. DEG enrichment in KEGG pathways was assessed using the software KOBAS [75]. We used the whole genome as the default background distribution. For each pathway that occurred in the set of genes, we counted the total number of genes in the set that were involved in the pathway. We then calculated the *p*-value using a hypergeometric distribution. We performed an FDR correction for multiple testing with default cut-off of 0.05.

### qRT-PCR validation

We randomly chose 15 differentially-expressed mRNAs and ten miRNAs for quantitative real-time RT-PCR (qRT-PCR) validation. The total RNA extraction procedure was as described above. For mRNA quantification, SuperScript™II Reverse Transcriptase (Invitrogen, USA) and Oligo(dT) primers were used to synthesise first-strand cDNA. Gene-specific primers were designed (Additional file 1: Table S1). A Bio-Rad CFX96 Real-time System was used for qRT-PCR. Three technical replicates were performed, and the BnActin7 gene was used as a control. SYBR® Green PCR Supermix (CA, USA) was chosen for qRT-PCR: the reactions contained 2 μL of cDNA, 10 μL of SYBR Supermix, 7.2 μL of H_2_O, and 0.4 μL primer (10 μM) in a volume of 20 μL. The reaction program was as follows: 98 °C for 30 s, followed by 40 cycles of 98 °C for 10 s and 60 °C for 30 s. For mature miRNA quantification, total miRNA was extracted from the serum samples by a miRcute Serum/Plasma miRNA Isolation Kit (TIANGEN, Beijing, China) according to the manual. Single-stranded cDNA was obtained from the extracted miRNA using the One Step Primer Script® miRNA cDNA Synthesis Kit (Takara) and SuperScript III Reverse Transcriptase (Invitrogen). qRT-PCR with three technical replicates for each reaction was performed to verify the expression of miRNAs through a miDETECT TractTM miRNA qRT-PCR Starter Kit (RiboBio, Guangzhou, China), using U6 snRNA of *B. napus* as a control. miRNA primers were showed in Additional file 1: Table S1. The 20 μl reaction consisting of 2 μl cDNA, 0.2 μM forward and reverse primer, and 10 μl of 2× SYBR Green PCR Master Mix, were carried out on an iCycler iQ real-time PCR detection system (BIO-RAD) with program as follows: 95 °C for 30 s, and 45 cycles of 95 °C for 5 s, 58 °C for 15 s, and 72 °C for 20 s.

## Supplementary information

**Additional file 1: Table S1.** The primer pairs for quantitative real-time RT-PCR (qRT-PCR) validation. **Table S2.** The statistics of sRNA-seq reads and mapped reads in shoot and root of oilseed rape seedlings under control condition and Cd stress. **Table S3.** The sequences of newly predicted miRNAs identified in 12 small RNA libraries. **Table S4.** The statistics of mRNA-seq reads and mapped reads in shoot (S1-S3) and root (R1-R3) tissues of oilseed rape seedlings under control condition (C) and Cd stress (T). **Table S5.** The significantly enriched GO terms during the cadmium response process in root and shoot of *B. napus*. **Table S6.** The differentially expressed genes related to the process of Cd transport in root and shoot of rapeseed seedling response to Cd treatment. **Table S7.** The differentially expressed genes related to the process of transcription regulation in root and shoot of rapeseed seedling response to Cd treatment. **Table S8.** The differentially expressed genes related to the process of secondary metabolism in root and shoot of rapeseed seedling response to Cd treatment. **Table S9.** The differentially expressed genes involved in Cd-induced antioxidant response in root and shoot of rapeseed seedling. **Table S10.** The differentially expressed genes involved in chlorophyll synthesis and photosynthesis in root and shoot of rapeseed seedling for Cd response. **Table S11.** The list of mRNA targets predicted for differentially expressed miRNAs in root and shoot.

**Additional file 2: Figure S1.** The expression pattern comparison between sequencing data and quantitative RT-PCR data for *Brassica napus* seedlings exposed to cadmium stress. **a** The comparison of differentially expressed mRNAs in root with the expression data from quantitative RT-PCR. **b** The comparison of differentially expressed miRNAs in root with the expression data from quantitative RT-PCR. **c** The comparison of differentially expressed mRNAs in shoot with the expression data from quantitative RT-PCR. **c** The comparison of differentially expressed mRNAs in root with the expression data from quantitative RT-PCR. **d** The comparison of differentially expressed miRNAs in shoot with the expression data from quantitative RT-PCR.

## Data Availability

The datasets generated and analyzed during the current study are presented within the manuscript and supplementary files. All raw sequence reads of both RNA-seq and small RNA-seq have been deposited in NCBI’s SRA database (https://trace.ncbi.nlm.nih.gov/Traces/sra/) and are accessible through SRA accession number PRJNA590544 (https://dataview.ncbi.nlm.nih.gov/object/PRJNA590544).

## Abbreviations

2-OG dioxygenase: 2-oxoglutarate dioxygenase
ABC: ATP Binding Cassette
APS: Adenosine phosphosulfate
CAM: Calmodulin
CAX: Cation/proton exchanger
CCS: Copper-zinc superoxide dismutase copper chaperone
Cd: Cadmium
CML: Calcium-binding proteins
COPT: Copper transport protein
CSD: Copper/zinc superoxide dismutase
CuAO: Copper amine oxidase
DEG: Differentially expressed gene
FDR: False discovery rate
FNR: Ferredoxin:NADP(H) oxidoreductase
GO: Gene ontology
GPOX: Glutathione peroxidase
GSH: Glutathione
GST: Glutathione S-transferase
KEGG: Kyoto Encyclopedia of Genes and Genomes
MiRNAs: MicroRNAs
MO: Monooxygenase
NIR: Nitrite reductase
NRT: Nitrate transporters
PAGE: Polyacrylamide gel electrophoresis
PC: Phytochelatin
POD: Peroxidase
PPase: Pyrophosphatase
RNA: Ribonucleic acid
ROS: Reactive oxygen species
SOD: Superoxide dismutase
TFs: Transcription factors
YSL: Yellow Stripe 1-like transporters
ZBDH: Zinc-binding dehydrogenase
ZIP: ZRT/IRT-like Proteins
