# Supplementary material for: MicroRNA-mRNA expression profiles and their potential role in cadmium stress response in Brassica napus
Source: BMC Plant Biol. 2019 Dec 19;19:570. doi: 10.1186/s12870-019-2189-9 (PMC6923997; doi:10.1186/s12870-019-2189-9)
Supplement: Supplementary file 2 — Additional file 2: Figure S1. The expression pattern comparison between sequencing data and quantitative RT-PCR data for Brassica napus seedlings exposed to cadmium stress. a The comparison of differentially expressed mRNAs in root with the expression data from quantitative RT-PCR. b The comparison of differentially expressed miRNAs in root with the expression data from quantitative RT-PCR. c The comparison of differentially expressed mRNAs in shoot with the expression data from quantitative RT-PCR. c The comparison of differentially expressed mRNAs in root with the expression data from quantitative RT-PCR. d The comparison of differentially expressed miRNAs in shoot with the expression data from quantitative RT-PCR. [file 12870_2019_2189_MOESM2_ESM.doc]

[MicroRNAs-mRNAs Expression Profile and Their Potential Role in](http://xueshu.baidu.com/s?wd=paperuri%3A(792c0f5ec370471760917439ed10f0c9)&filter=sc_long_sign&sc_ks_para=q%3DMicroRNAs-mRNAs Expression Profile and Their Potential Role in Malignant Transformation of Human Bronchial Epithelial Cells Induced by Cadmium.&sc_us=14390948532090839949&tn=SE_baiduxueshu_c1gjeupa&ie=utf-8) Cadmium Stress Response in *Brassica napus*

Ying Fu**^1^, Annaliese S. Mason^2^, Yaofeng Zhang^1^, Baogang Lin****^1^, Donghui Fu^3^* and Huasheng Yu^1^***

^1^Institute of Crop and Nuclear Technology Utilization, Zhejiang Academy of Agricultural Sciences, Hangzhou, China

^2^Department of Plant Breeding, IFZ for Biosystems, Land Use and Nutrition, Justus Liebig University, Heinrich-Buff-Ring 26-32, 35392 Giessen, German

^3^Key Laboratory of Crop Physiology, Ecology and Genetic Breeding, Ministry of Education, Agronomy College, Jiangxi Agricultural University, Nanchang 330045, China.

*Corresponding author

Telephone: +086-0791-83813142;

Fax: +086-0791-83813185

E-mail: [fudhui@163.com](mailto:fudhui@163.com);

or Phone: +86 (0) 571 86404096

Fax: +86 (0) 571 86404096

E-mail: yuhuasheng-0@163.com

**Figure S1 The expression pattern comparison between sequencing data and quantitative RT-PCR data for *Brassica napus* seedlings exposed to cadmium stress. a** The comparison of differentially expressed mRNAs in root with the expression data from quantitative RT-PCR. **b** The comparison of differentially expressed miRNAs in root with the expression data from quantitative RT-PCR. **c** The comparison of differentially expressed mRNAs in shoot with the expression data from quantitative RT-PCR. **c** The comparison of differentially expressed mRNAs in root with the expression data from quantitative RT-PCR. **d** The comparison of differentially expressed miRNAs in shoot with the expression data from quantitative RT-PCR.
